# Effect of Surface Morphology Formed by Additive Manufacturing on the Adhesion of Dental Cements to Zirconia

**DOI:** 10.3390/ma19030563

**Published:** 2026-01-31

**Authors:** Kumiko Yoshihara, Noriyuki Nagaoka, Sungho Lee, Yukinori Maruo, Fiona Spirrett, Soshu Kirihara, Yasuhiro Yoshida, Bart Van Meerbeek

**Affiliations:** 1 National Institute of Advanced Industrial Science and Technology (AIST), Health and Medical Research Institute, Takamatsu 761-0395, Japan; 2 Department of Pathology & Experimental Medicine, Graduate School of Medicine, Dentistry and Pharmaceutical Sciences, Okayama University, Okayama 700-8558, Japan; 3 Advanced Research Center for Oral and Craniofacial Sciences, Okayama University Dental School, Okayama 700-8558, Japan; nagaoka@okayama-u.ac.jp; 4 National Institute of Advanced Industrial Science and Technology (AIST), Nagoya 463-8560, Japan; sungho.lee@aist.go.jp; 5 Department of Prosthodontics, Okayama University, Okayama 700-8558, Japan; ykmar@md.okayama-u.ac.jp; 6 Joining and Welding Research Institute, Osaka University, Osaka 567-0047, Japankirihara.soshu.jwri@osaka-u.ac.jp (S.K.); 7 Department of Biomaterials and Bioengineering, Faculty of Dental Medicine, Hokkaido University, Sapporo 060-8586, Japan; yasuhiro@den.hokudai.ac.jp; 8 Department of Oral Health Sciences, BIOMAT, KU Leuven, 3000 Leuven, Belgium; bart.vanmeerbeek@kuleuven.be

**Keywords:** additive manufacturing, bond strength, dental crown, dental resin cement, dental zirconia

## Abstract

Background: Durable bonding to zirconia remains difficult because its chemically inert surface resists acid etching. Additive manufacturing (AM) enables controlled surface morphology, which may enhance micromechanical retention without additional treatments. Methods: Zirconia specimens with three AM-derived surface designs—(1) concave–convex hemispherical patterns, (2) concave hemispherical patterns, and (3) as-printed surfaces—were fabricated using a slurry-based 3D printing system and sintered at 1500 °C. Zirconia specimens fabricated by subtractive manufacturing using CAD/CAM systems, polished with 15 µm diamond lapping film and with or without subsequent alumina sandblasting, served as controls. Surface morphology was analyzed by FE-SEM, and shear bond strength (SBS) was tested after cementation with a resin-based luting agent. Results: SEM revealed regular layered textures and designed hemispherical structures (~300 µm) in AM specimens, along with step-like irregularities (~40 µm) at layer boundaries. The concave–convex AM group showed significantly higher SBS than both sandblasted and polished subtractive-manufactured zirconia (*p* < 0.05). Vertically printed specimens demonstrated greater bonding strength than those printed parallel to the bonding surface, indicating that build orientation affects resin infiltration and interlocking. Conclusion: AM-derived zirconia surfaces can provide superior and reproducible micromechanical retention compared with conventional treatments. Further optimization of printing parameters and evaluation of long-term durability are needed for clinical application.

## 1. Introduction

In modern dentistry, there is a rapidly increasing demand for restorative materials that combine esthetics, durability, and biocompatibility [1]. Concerns over the high cost and limited biocompatibility of metals have driven a significant shift toward metal-free restorative options [2,3]. As a result, tooth-colored materials such as composite resins, glass ceramics, and zirconia have become widely accepted in clinical practice [4]. Advances in computer-aided design and computer-aided manufacturing (CAD/CAM) technologies have further facilitated the fabrication of these restorative materials [5,6]. Among them, zirconia has attracted considerable attention because of its superior strength and favorable biocompatibility, making it an optimal material for fixed partial dentures and implant-supported prostheses [7].

Despite these advantages, achieving reliable bonding between zirconia and resin-based cements remains a clinical challenge [8]. To enhance adhesion, the use of functional monomers such as 10-methacryloyloxydecyl dihydrogen phosphate (10-MDP) and silane coupling monomer 3-methacryloxypropyltrimethoxysilane (γ-MPTS) in combination with mechanical retention methods, including alumina sandblasting, has been recommended. However, the blasting pressure applied to zirconia is weaker than that used for metals, and thus the mechanical interlocking achieved is often insufficient [9]. Considering the long-term stability of adhesion, it is clinically important to establish more effective methods for creating durable micromechanical retention on zirconia surfaces.

As an alternative to subtractive manufacturing using CAD/CAM processing, additive manufacturing (AM), also known as three-dimensional (3D) printing, offers a novel approach for fabricating ceramic restorations [10]. Unlike milling, which wastes most of the original block, AM builds structures layer by layer, improving both design flexibility and material efficiency [11]. Recent advances in ceramic slurry-based AM systems have enabled the production of sintered zirconia with high density and promising mechanical properties [10,12]. Importantly, AM allows for the intentional design of unique internal topographies that cannot be achieved by conventional subtractive manufacturing techniques [13].

However, despite these technological advances, current research on additively manufactured zirconia has predominantly focused on a broad range of topics such as mechanical properties, dimensional accuracy, and restoration fit. In contrast, studies addressing adhesive performance remain relatively limited. Although several reports have investigated the effect of designed surface morphologies produced by AM on zirconia–resin bonding, the influence of layer-induced surface steps inherent to the additive manufacturing process has not been sufficiently explored. In particular, there is a lack of systematic comparison between the bonding performance achieved by the random surface roughness generated by alumina sandblasting, which is currently the recommended clinical approach for CAD/CAM zirconia, and that achieved by intentionally designed AM surface microstructures combined with the intrinsic anisotropy introduced by layer-by-layer fabrication. Consequently, the relative contribution of AM-specific surface design and build-induced anisotropy to zirconia–resin bond strength remains largely unclear.

Therefore, the hypothesis of this study was that intentionally designed AM-derived surface microstructures with controlled build orientation would provide superior and more reproducible micromechanical retention compared with conventional sandblasted zirconia surfaces. The objective of the present study was to fabricate zirconia specimens with different surface morphologies using additive manufacturing, to compare their microstructural characteristics with those of conventionally manufactured zirconia produced by subtractive manufacturing, and to evaluate the effect of surface design and build orientation on shear bond strength to resin cement.

## 2. Materials and Methods

### 2.1. Specimen Preparation and Experimental Groups

**Figure 1:**
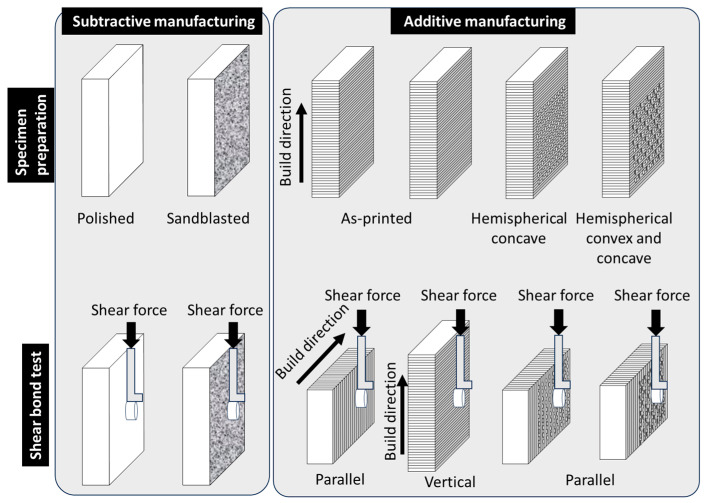
Schematic diagram of the study design.

In this study, three-dimensional fabrication was performed using a top-down stereolithography system [12]. A 3Y-TZP zirconia resin slurry (SK Fine, Kusatsu, Japan) was layer-built with a thickness of 50 µm using a stereolithography (SZ1100, SK Fine), and each layer was photopolymerized to form the structure. The slurry composition, printing parameters, debinding process, and sintering conditions were identical to those described in our previous study [12]. As shown in Figure 1, the specimens were designed so that the shear bond test surface was oriented perpendicular (90°) to the build direction.

Three types of rectangular plates (5 × 10 × 2.1 mm) were fabricated:

(1) A surface with alternating hemispherical convex and concave features (diameter: 0.3 mm, spacing: 0.5 mm), possessing both convex and concave characteristics;
(2) A surface with only hemispherical concave features (diameter: 0.3 mm, spacing: 0.5 mm); and
(3) A surface without any intentional surface pattern (As-printed surface).

For each additively manufactured experimental group, ten plates were independently fabricated (*n* = 10), with each plate representing one independent specimen. After printing, all specimens were ultrasonically cleaned in ethanol, and subjected to debinding and sintering under the same conditions as reported previously [12], debinding, and sintering at 1500 °C.

For comparison, zirconia specimens fabricated by subtractive manufacturing using CAD/CAM systems were also prepared as independent specimens (*n* = 10 per group). Subtractive-manufactured zirconia plates (Katana HT, A2, Kuraray Noritake Dental Inc., Tokyo, Japan) with dimensions of 10 × 10 × 2 mm were sintered to final density at 1500 °C according to the manufacturer’s instructions. These specimens were then divided into two surface treatment groups: a polished group and a sandblasted group.

For the polished group, the zirconia surfaces were finished using 15 µm diamond lapping film (3 M). For sandblast group, the surface was treated with alumina sandblasting (particle size: 15 μm) using sandblaster (Hiblaster Ovarjet, Shofu, Kyoto, Japan) at 0.4 MPa for 5 s at a distance of 10 mm. A schematic diagram of the experimental procedure used in this study is shown in Figure 1.

### 2.2. SEM Observation

The surface of each specimen was coated with a thin layer of osmium using an osmium plasma coater (Neoc-STB, Meiwafosis, Tokyo, Japan) to provide conductivity. The coated specimens were examined using a field-emission scanning electron microscope (FE-SEM; JSM-6701F, JEOL, Tokyo, Japan). Photomicrographs of the bonding surfaces were acquired for morphological analysis.

### 2.3. Sample Preparation for Shear Bond Strength (SBS)

The specimens were washed with ethanol, subsequently irradiated with a Xe_2_ excimer lamp (UER20-172B, Ushio Electric, Tokyo, Japan) at an ultraviolet (UV) wavelength (λ = 172 nm) in ambient air to clean the surface. Sintered zirconia round bars (3.4 mm diameter, 3 mm thickness) were prepared by Tosoh (Tokyo, Japan). The zirconia round bars were sandblasted (Hiblaster Ovaljet, Shofu, Kyoto, Japan) with 50 µm alumina particles (Hi aluminas, Shofu) at 0.3 MPa for 5 s at a distance of 10 mm to ensure that the cement bonded strongly to the sandblasted zirconia surface and would not fail during the SBS test. Each plate (*n* = 10) was provided. The sandblasted zirconia bars were luted onto each specimen (one bar per plate) with Panavia V5 (Kuraray Noritake Dental Inc., Tokyo, Japan) using finger pressure (corresponding to a pressure of about 2.2 MPa). The resin-based composite cement was cured for 1 min using a G-Light Prima II Plus lamp (2800 mW/cm^2^ light irradiance; GC Corporation, Tokyo, Japan) [14].

The specimens were mounted on a material-testing machine (Model 5565, Instron, Canton, MA, USA). In the additively manufactured groups, the mount orientation of the specimens was predetermined. For the hemispherical concave and hemispherical concave and convex specimens, shear stress was applied parallel to the printing layers. In the as-printed specimens, two types of shear loading were applied: one parallel and the other perpendicular to the printing layers. The shear stress was applied at a cross-head speed of 0.5 mm/min. After SBS testing, all failed specimens were analyzed using a light microscope (40×) (SMZ-10, Nikon, Tokyo, Japan) to assess the fracture pattern, which was used to classify the samples as having failed “cohesively”, “adhesively”, or “mixed” (involving both cohesive and adhesive failure regions). For statistical analysis, one-way analysis of variance (ANOVA) followed by Tukey’s multiple comparison test was used with α = 0.05.

## 3. Results

### 3.1. SEM Observation of Zirconia Surface

**Figure 2:**
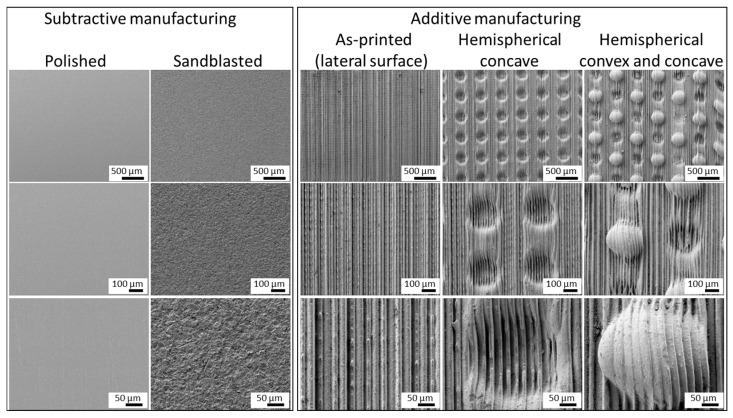
SEM images at various magnifications of subtractive and additively manufactured zirconia tested in this study.

The representative surface morphologies of zirconia specimens with different fabrication methods and surface treatments were observed using scanning electron microscopy (SEM) (Figure 2). The untreated subtractive group exhibited a relatively smooth surface with faint machining marks and small pits visible at higher magnification. After sandblasting, the surface showed pronounced roughness with irregular asperities and fractured features, indicating the formation of microretentive topography.

In contrast, the additively manufactured specimens displayed distinct surface features corresponding to the printing process. In the as-printed condition, step-like features approximately 40 µm in width were observed, which originated from the 50 µm printing layers that shrank during sintering. The layered structures were not entirely uniform and exhibited slight irregularities. In the hemispherical concave samples, similar layered patterns were observed, along with regularly arranged concave features. The magnified images revealed hemispheres with a diameter of approximately 300 µm. In the hemispherical concave and convex specimens, layer-like patterns similar to those in the as-printed samples were also evident, together with clearly visible regularly arranged concave and convex textures. Furthermore, both concave and convex hemispherical structures with diameters of approximately 300 µm were well formed, and distinct layer boundaries remained within these features. The dimensions of the surface features reported in this study correspond to post-sintering values. SEM observation confirmed that the designed hemispherical geometries and layer-induced surface structures were retained after debinding and sintering, despite an overall linear shrinkage of approximately 20%.

### 3.2. Shear Bond Strength Test

**Figure 3:**
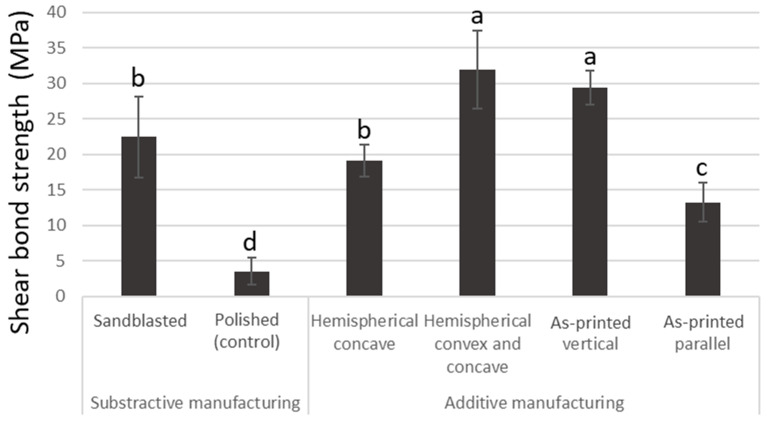
Biaxial flexural strength of zirconia fabricated by additive and subtractive manufacturing methods. Error bars represent standard deviations (SDs). Means with the same letter are not significantly different from each other (*p* > 0.05).

The shear bond strengths of zirconia specimens with different surface treatments and fabrication methods are shown in Figure 3. Among the subtractive groups, the sandblasted specimens exhibited significantly higher bond strength than the untreated polished (control) group. In the additively manufactured groups, the bond strength varied depending on the printing direction and surface geometry. The hemispherical concave specimens showed moderate bond strength, whereas the hemispherical concave and convex specimens demonstrated the highest bond strength. The printing direction also affected bonding performance: specimens printed in the vertical direction exhibited higher bond strength than those printed in the parallel direction, where shear stress was applied along the printing layers. Accordingly, the hemispherical concave and hemispherical concave and convex specimens showed higher bond strength compared with those without any surface geometry. Failure mode evaluation revealed distinct differences among the experimental groups. The polished subtractive-manufactured zirconia specimens predominantly showed adhesive failure at the zirconia–cement interface, indicating insufficient interfacial bonding. In contrast, all other groups, including sandblasted subtractive-manufactured zirconia and all additively manufactured specimens, exhibited cohesive failure within the resin cement. No interfacial debonding between zirconia and cement was observed in these groups.

Statistical analysis using one-way ANOVA followed by Tukey’s HSD test revealed significant differences among the groups (F(5, 54) = 78.56, *p* < 0.05). The hemispherical concave and convex and as-printed parallel additive-manufactured specimens exhibited significantly higher bond strength than both the sandblasted subtractive specimens and the other additive manufacturing conditions. The untreated polished group (control) and the as-printing parallel group showed the lowest bond strengths, which were significantly different from all other groups (*p* < 0.05).

## 4. Discussion

In this study, the influence of surface morphology formed by additive manufacturing (AM) on the shear bond strength of zirconia was investigated. Zirconia possesses high mechanical strength and chemical stability but cannot be etched with hydrofluoric acid, unlike glass ceramics. Therefore, mechanical interlocking is typically achieved by sandblasting [15]. However, excessive blasting pressure can induce surface flaws or microcracks in zirconia, leading to potential degradation of long-term bond durability. In contrast, AM enables precise control of surface morphology during fabrication, allowing the production of microstructured surfaces without the need for post-processing [16]. This study aimed to clarify the effect of such AM-derived microstructures on the adhesive performance of zirconia, focusing on mechanical interlocking rather than chemical adhesion. Many resin cement systems, such as PANAVIA SA Cement Universal (Kuraray Noritake Dental Inc., Japan), contain 10-MDP directly in the cement paste, enabling chemical bonding to zirconia during cementation. In contrast, the resin cement paste of Panavia V5 does not contain 10-MDP. In the Panavia V5 system, chemical adhesion to substrates is achieved through separate primers: Panavia V5 Tooth Primer for tooth structures and Clearfil Ceramic Primer Plus (Kuraray Noritake Dental Inc., Japan) for zirconia and other ceramic materials, both of which contain 10-MDP. In the present study, Clearfil Ceramic Primer Plus was intentionally not applied in order to exclude the influence of chemical adhesion and to specifically evaluate the effect of surface morphology-induced micromechanical interlocking on zirconia–resin bonding.

SEM revealed that the AM zirconia surface exhibited characteristic topographical features depending on the printing direction. The stacking of 50 µm thick zirconia slurry layers, followed by debinding and sintering, resulted in approximately 20% shrinkage, forming step-like irregularities of around 40 µm at the layer boundaries. These layer ends produced hemispherical concavities approximately 300 µm in diameter. The shear bond strength test demonstrated that the printing orientation had a significant effect on adhesive performance. Specimens printed with the building layers perpendicular to the bonding surface showed significantly higher bond strength than those printed parallel to the bonding surface. This difference can be attributed to the enhanced resin penetration into the vertically oriented microsteps, which promotes stronger micromechanical interlocking. Conversely, parallel printing may restrict effective penetration due to alignment of the interface and layering direction. These findings highlight the importance of anisotropy inherent in AM processes and suggest that optimizing printing orientation could enhance interfacial bonding performance. In the present study, shear bond strength testing of specimens with intentionally designed concave or concave–convex surface morphologies was performed only in the orientation parallel to the build surface. This orientation was deliberately selected to minimize the influence of layer-induced step irregularities inherent to the additive manufacturing process and to allow the effect of the designed surface morphology itself to be evaluated more directly. In contrast, for as-printed specimens without intentional surface design, both parallel and perpendicular orientations were examined in order to assess the anisotropic effect of build direction on bonding performance. By adopting this experimental strategy, the influence of designed surface topography on micromechanical interlocking could be isolated without confounding effects arising from build-orientation-dependent surface irregularities.

Even without chemical surface treatment such as silane or ceramic primers, certain AM zirconia specimens exhibited bond strengths significantly higher than conventionally sandblasted subtractive-manufactured zirconia. This suggests that precisely designed and uniformly distributed microstructures produced by AM can provide superior and reproducible micromechanical retention compared with the random roughness induced by sandblasting. Moreover, because AM enables surface morphology to be introduced during fabrication, additional chairside surface treatment is unnecessary. This could reduce clinical operation time and potential errors during sandblasting. Furthermore, avoiding sandblasting eliminates the risk of surface damage such as microcracks and chipping, thereby improving material integrity. Collectively, these results indicate that AM is a promising approach for fabricating zirconia bonding surfaces with both mechanical reliability and clinical practicality.

The present findings are consistent with previous reports investigating the effects of surface geometry on zirconia bonding. Dai et al. evaluated groove (width and depth of 0.4 mm and 0.09 mm, respectively) and hexagonal grid textures (side length 0.4 mm, depth 0.09 mm) and reported that sandblasting further enhanced their bond strength [17]. Liu et al. examined surface textures of various shapes (circle, triangle, square, pentagon, and hexagon) and diameters (400 µm, 800 µm) and found that circular patterns provided significantly higher immediate shear bond strength (SBS) than triangular ones (*p* < 0.01), while other shapes showed no significant differences (*p* > 0.05) [18]. Zandinejad et al. compared designed porosities of 50 × 50 µm, 100 × 100 µm, and 200 × 200 µm spaced at 200, 400, and 800 µm, respectively, and demonstrated that the largest pores (200 µm) achieved comparable strength to sandblasted controls, whereas smaller pores resulted in significantly lower values [19]. Taken together, these studies indicate that adhesive performance depends strongly on the shape, size, and orientation of the designed surface structures. The present results support this view, demonstrating that microstructures fabricated via AM can provide an intentionally optimized micromechanical interlocking interface comparable or superior to that produced by conventional methods.

Future studies should focus on the systematic optimization of design parameters such as geometry, dimension, and layer orientation, as well as on evaluating the long-term durability of AM zirconia interfaces under simulated intraoral conditions. Since the present study was limited to static shear testing, further research incorporating dynamic loading or thermocycling aging is required to assess the mechanical stability of the adhesive interface in the oral environment. Although clinical data on AM zirconia restorations remain limited, the ability to tailor surface morphology with high precision suggests that AM zirconia represents a promising next-generation dental material combining strength, esthetics, and optimized adhesion.

## 5. Conclusions

In this study, the relationship between the surface morphology of additively manufactured zirconia and the initial bonding strength of resin cement was investigated, with a particular focus on micromechanical interlocking. The results demonstrated that AM-derived surface microstructures significantly influence bonding performance. In particular, irregularities formed at layer boundaries enhanced mechanical interlocking compared with conventional sandblasting or laser treatments.

These findings indicate that additive manufacturing enables novel surface designs that are not achievable by traditional fabrication methods and can effectively improve initial zirconia–resin bonding through mechanical means. However, the present results are limited to short-term bonding performance and do not address long-term durability under simulated oral conditions. Therefore, further optimization of surface design parameters, as well as comprehensive evaluation including thermocycling, hydrolytic aging, and dynamic loading tests, is required before clinical applicability can be established.

## Data Availability

The data that support the findings of this study are available from the corresponding author, K.Y., upon reasonable request.
